# Prevalence and risk factors of malaria in Ethiopia

**DOI:** 10.1186/1475-2875-11-195

**Published:** 2012-06-12

**Authors:** Dawit G Ayele, Temesgen T Zewotir, Henry G Mwambi

**Affiliations:** 1 School of Mathematics, Statistics and Computer Science, University of KwaZulu-Natal, Pietermaritzburg, Private Bag X01, Scottsville, 3209, South Africa

**Keywords:** Generalized linear model, Odds ratio, Rapid diagnosis test, Risk factors, Survey design

## Abstract

**Background:**

More than 75% of the total area of Ethiopia is malarious, making malaria the leading public health problem in Ethiopia. The aim of this study was to investigate the prevalence rate and the associated socio-economic, geographic and demographic factors of malaria based on the rapid diagnosis test (RDT) survey results.

**Methods:**

From December 2006 to January 2007, a baseline malaria indicator survey in Amhara, Oromiya and Southern Nation Nationalities and People (SNNP) regions of Ethiopia was conducted by The Carter Center. This study uses this data. The method of generalized linear model was used to analyse the data and the response variable was the presence or absence of malaria using the rapid diagnosis test (RDT).

**Results:**

The analyses show that the RDT result was significantly associated with age and gender. Other significant covariates confounding variables are source of water, trip to obtain water, toilet facility, total number of rooms, material used for walls, and material used for roofing. The prevalence of malaria for households with clean water found to be less. Malaria rapid diagnosis found to be higher for thatch and stick/mud roof and earth/local dung plaster floor. Moreover, spraying anti-malaria to the house was found to be one means of reducing the risk of malaria. Furthermore, the housing condition, source of water and its distance, gender, and ages in the households were identified in order to have two-way interaction effects.

**Conclusion:**

Individuals with poor socio-economic conditions are positively associated with malaria infection. Improving the housing condition of the household is one of the means of reducing the risk of malaria. Children and female household members are the most vulnerable to the risk of malaria. Such information is essential to design improved strategic intervention for the reduction of malaria epidemic in Ethiopia.

## Background

Malaria is a life-threatening caused by *Plasmodium* parasite infection. Malaria is the most deadly, and it predominates in Africa [1]. The problem of malaria is very severe in Ethiopia where it has been the major cause of illness and death for many years [1,2]. According to records from the Ethiopian Federal Ministry of Health, 75% of the country is malarious with about 68% of the total population living in areas at risk of malaria [1,2]. That is, more than 50 million people are at risk from malaria [3], and four to five million people are affected by malaria annually [4,5]. The transmission of malaria in Ethiopia depends on altitude and rainfall with a lag time varying from a few weeks before the beginning of the rainy season to more than a month after the end of the rainy season [6,7]. Epidemics of malaria are relatively frequent [8,9] involving highland or highland fringe areas of Ethiopia, mainly areas 1,000-2,000 m above sea level [1,7,10]. Malaria transmission peaks bi-annually from September to December and April to May, coinciding with the major harvesting seasons. This has serious consequences for Ethiopia’s subsistence economy and for the nation in general. Major epidemics occur every five to eight years with focal epidemics as the commonest form. Early diagnosis and prompt treatment is one of the key strategies in controlling malaria. For areas where laboratory facilities are not available, clinical diagnosis is widely used [11,12]. To diagnose malaria, microscopy remains the standard method, but it is not accessible or affordable in most peripheral health facilities. The recent introduction of rapid diagnostic tests (RDT) for malaria is a significant step forward in case detection, management and reduction of unnecessary treatment. RDT could be used in malaria diagnosis during population-based surveys and to provide immediate treatment based on the results.

Rapid diagnostic tests (RDTs) for malaria offer the potential to extend accurate malaria diagnosis to areas when microscopy services are not available, such as in remote locations or after regular laboratory hours. Rapid malaria diagnostic tests have been developed in the lateral flow format [13]. These tests use finger-stick blood, take only 10 to 15 minutes to complete, and do not require a laboratory. Non-clinical staff can easily learn to perform the test and interpret the results [14]. The objective of this paper is to identify the socio-economic, geographic and demographic risk factors of malaria using the rapid diagnosis test (RDT).

## Methods

### Study design

A baseline household cluster malaria survey was conducted by The Carter Center from December 2006 to January 2007. A questionnaire was developed as a modification of the Malaria Indicator Survey (MIS) Household Questionnaire. The questionnaire had two parts; the household interview and malaria parasite form. For this survey, the sampling frame was the rural populations of Amhara, Oromiya and SNNP regions, which is *kebele* (the smallest administrative unit in Ethiopia). Firstly, 224 *kebeles* of 25 household each were selected. From each *kebele*, out of the 25 households 12 even-numbered households were selected for malaria tests. All members of the household were tested for malaria by using RDT. In the survey, each room in the house was listed separately. During the study period, 5,708 households which were located in 224 clusters, covered in the survey. From the total of 5,708 households, Amhara, Oromiya and SNNP regions cover 4,101 (71.85%), 809 (14.17%) and 798 (13.98%) households respectively [15].

For the baseline household cluster malaria survey which was conducted by The Carter Center, a multi-stage cluster random sampling was used. By assuming the lowest measurement of prevalence malaria indicator, the sample size was estimated. Based on the assumption that prevalence of malaria to be the lowest indicator to be measured, the prevalence in the population was taken to be 8%. In Amhara region, each zone was regarded as a separate domain, while in Oromiya and SNNPR, the community-directed treatment with ivermectin (CDTI) areas combined were one domain. All ten Amhara zones were surveyed as separate domains, with 16 clusters in each zone (total 160 clusters). Bahir Dar town and two *woredas* with less than 10% of the population living in malarious areas were excluded. In Oromiya and SNNPR, sampling was done directly at the *kebele* level. From the total number of individuals who participated in the survey, 7,745 in Amhara, 1,996 in Oromiya and 1,860 in SNNP from all age groups were tested using RDT [15]. Further studies on the sampling procedure for the survey were studied by different researchers [16,17].

Malaria parasite testing was performed on consenting residents. A blood sample was collected by taking finger-prick blood from participants for malaria RDT. The test is capable of detecting both *Plasmodium falciparum* and other *Plasmodium* species. Participants with positive rapid tests were immediately offered treatment according to national guidelines.

Using the baseline household cluster malaria survey which was conducted by The carter Center in Amhara, Oromiya and SNNP regions, a number of research papers have been published. Individual, household and environmental risk factors of malaria in Amhara, Oromiya and SNNP regions of Ethiopia was studied by Graves *et al.* in 2008 [18]. To assess malaria infections in relation to socio-economic, demographic and environmental factors, they used univariate analysis. From the result it can be seen that overall prevalence of malaria was found to be low. The detailed report for this survey is presented by The Carter Center [15]. The other research paper which was conducted using this population-based survey is evaluation of light microscopy and rapid diagnosis test. This was done by Endeshaw *et al.* in 2008 [19]. The finding of this study suggested that blood slide microscopy found to be the best option for population-based prevalence survey of malaria *parasitaemia*. Similarly, Sharge *et al.* studied net coverage in Oromiya and SNNP regions of Ethiopia and ownership and use of long lasting insecticidal nets in 2008 and 2010 [17,20]. The result from these studies implies that malaria continues to be a significant public health problem in the surveyed regions of Ethiopia. The use of mosquito nets resulted in the decline of the prevalence of malaria in Amhara, Oromiya and SNNP regions of Ethiopia. These studies focused only to univariate analysis, but advanced statistical analysis is very important to identify the socio-economic, demographic and geographic factors which have influence to the risk of malaria. Multivariate statistical methods used for this study. Therefore, in this study the variables of interest are as follows.

### Response variable

The outcome of interest is malaria RDT result. RDTs assist in the diagnosis of malaria by detecting evidence of malaria parasites in human blood and are an alternative to diagnosis based on clinical grounds or microscopy, particularly where good quality microscopy services cannot be readily provided. Thus, the response variable is binary, indicating whether or not a person was positive for malaria.

### Independent variables

The independent covariates comprised the baseline socio-economic, demographic, and geographic variables that included gender, age, family size, region, altitude, main source of drinking water, time taken to collect water, toilet facilities, availability of electricity, radio and television, total number of rooms, main material of the room's wall, main material of the room's roof, main material of the room's floor, incidence of anti-malarial spraying in the past 12 months, use of mosquito nets and total number of nets. Malaria test (RDT result), age and sex were collected at individual level. Altitude, main source of drinking water, time taken to collect water, toilet facilities, availability of electricity, radio, television, total number of rooms, main material of the room's walls, main material of the room's roof, main material of the room's floor, use of anti-malarial spray in the past 12 months, use of mosquito nets and total number of nets were all collected at household level.

### The statistical model

Data was analysed by fitting a generalized linear model (GLM). The GLM generalizes linear regression by relating the response variable to predictor variables via a link function and by allowing the magnitude of the variance of each measurement to be a function of its predicted value.

The class of GLM includes many well-known statistical models such as: multiple regression for normal responses; logistic and probit regression for binary responses; binomial counts, or proportions; Poisson and negative binomial regression; log-linear categorical data analysis models; gamma regression for variance models; and exponential and gamma models for survival time models.

The literature on GLM and their extensions is vast [21-24]. Generalized linear models have been extended in many ways, such as accommodating random and mixed effects, accommodating correlated data, relaxing distributional assumptions, allowing semi-parametric linear predictors [25,26].

The logistic regression model is classified under GLM. This model is used to model binary data. The logistic regression model used to analyse data from complex sampling designs is referred to as survey logistic regression models. Survey logistic regression models have the same theory as ordinary logistic regression models. The difference between ordinary and survey logistic is that survey logistic accounts for the complexity of survey designs. But, for data from simple random sampling, the survey logistic regression model and the ordinary logistic regression model are identical.

For ordinary logistic regression, a method of maximum likelihood estimation is used to estimate parameters of the model. But, estimation of the standard errors of the parameter estimates is very complicated for data that comes from complex designs. The complexities in variance estimation arise partly from the complicated sample design and the weighting procedure imposed. Therefore, the incorporation of sampling information is important for the proper assessment of the variance of a statistic [27-29]. Since weighting and specific sample designs are particularly implemented for increasing the efficiency of a statistic, their incorporation in the variance estimation methodology is of major importance [30]. Thus, the bias induced under this simplifying approach depends on the particular sampling design and should be investigated circumstantially. Therefore, there are several methods to obtain the covariance matrix [31]. These methods include the Taylor expansion approximation procedure, jack-knife estimator, bootstrap estimator, balanced repeated replication method and random groups method [32,33].

## Results

The data analysis for this study was done using SAS version 9.2. The deviance was used to compare alternative models during model selection. Change in the deviance was used to measure the extent to which the fit of the model improves when additional variables were included. To avoid confounding effects, the model was fitted in two steps. The model was fitted to each predictor variables one at a time. In stage two the significant predictors were retained in a multivariate logistic regression model. In addition to the main effects, possible combinations of up to three-way interaction terms were added and assessed to further avoid and mitigate the problem of confounding.

The objective of the analysis is to identify the individual characteristics that could be associated with the malaria rapid diagnosis test outcome. On the other hand, this study focused on identifying the household characteristics which could be associated with the increase/decrease of the number of malaria infected household members. These household characteristics which were included in the model are main source of drinking water, time taken to collect water, toilet facilities, availability of electricity, radio and television, number of persons per room, main material of the room's wall, main material of the room's roof, main material of the room's floor, use of anti-malaria spray in the past 12 months, use of mosquito nets, number of nets per person, family size, region and altitude of region. The individual characteristics are gender and age.

To make statistically valid inferences, the analysis of the data must account for the design of the study. The SAS procedure which performs logistic regression for categorical responses in sample survey data was used [34].

The maximal model with significant effects is given in Tables 1 and 2. These models have the smallest deviance (−2logL) amongst all the nested models with the three-way interaction effects. Based on the final model, six interactions reduced the deviance (−2logL). Therefore, the final model includes all the main effects and the six interaction effects.

**Table 1:** Estimates and odds ratios of socio-economic, demographic and geographic factors on RDT

	**Estimate**	**OR**	**95% CI**		**P -value**
			**Lower**	**Upper**	
Intercept	−3.030	0.048	0.016	0.125	0.001
Age	−0.031	0.970	0.319	2.505	0.0001
Sex (ref. male)					
Female	−1.820	0.162	0.053	0.418	<.0001
Family size	0.049	1.057	1.014	1.124	<.0001
Region (ref. SNNP)					
Amhara	−0.099	0.906	0.178	0.183	0.521
Oromiya	−0.184	0.832	0.238	8.581	0.183
Toilet facility (Ref. No facility)					
					
Pit latrine	−0.3213	0.725	2.575	2.147	<.0001
Toilet with flush	−0.5935	0.552	2.632	4.909	<.0001
Main source of drinking water (ref. protected water)					
Tap water	−0.038	0.963	0.316	0.373	<.0001
Unprotected water	0.717	2.048	0.673	5.289	0.007
Availability of television (ref. no)					
Yes	0.304	1.356	0.446	3.500	0.024
Number of rooms/person	−0.473	0.623	0.205	1.610	0.044
Main material of room's wall (ref. cement block)					
Mud block/stick/wood	−2.326	0.098	0.032	0.252	0.048
Corrugated metal	−0.620	0.538	0.471	0.826	0.001
Main material of room's roof (ref. corrugate)					
Thatch	1.325	3.761	1.236	9.712	<.0001
Stick and mud	−1.960	0.141	0.046	0.364	<.0001
Main material of room's floor (ref. earth/Local dung plaster)					
Wood	−1.701	0.183	0.149	0.443	<.0001
Cement	−3.927	0.014	1.014	4.876	0.018
Anti-malarial spraying					
No	1.857	6.405	2.105	16.539	0.046
Use of mosquito nets (ref. no)					
Yes	−0.095	0.910	0.299	2.349	<.0001
Number of nets/person	−0.782	0.457	0.150	1.181	<.0001

**Table 2:** Estimates and odds ratios of socio-economic, demographic and geographic factors on RDT for interaction effects

	**Estimate**	**OR**	**95% CI**		**P -value**
			**Lower**	**Upper**	
Main source of drinking water and main material of the room's roof (ref. Protected water & cement block)					
Tap water and Mud block/stick/wood	−3.339	0.035	0.007	0.177	<.0001
Tap water and Corrugated metal	−3.377	0.034	0.007	0.184	<.0001
Unprotected water and Mud block/stick/wood	−4.008	0.018	0.003	0.130	<.0001
Unprotected water and Cement block	−1.857	0.156	0.022	1.119	<.0001
Time to collect water and material of room's floor (ref. Less than 30 minutes and earth/local dung plaster)					
Greater than 90 minutes and Cement	−0.423	0.655	0.066	1.478	<.0001
Greater than 90 minutes and Wood	−0.721	0.486	0.160	1.478	0.0013
Between 30–40 minutes and Cement	−1.901	0.149	0.049	1.478	<.0001
Between 30–40 minutes and Wood	1.554	4.729	0.821	9.220	<.0001
Between 40–90 minutes and Cement	−0.739	0.933	0.129	1.258	0.0011
Between 40–90 minutes and Wood	0.554	3.769	1.835	7.232	<.0001
Gender and main source of drinking water and main material of the room's roof (ref. Male & protected water)					
Female and Tap water	−0.069	0.933	0.624	1.397	0.0972
Female and Unprotected water	1.327	3.769	1.948	7.293	<.0001
Gender and material of room's floor (ref. Male and earth/Local dung plaster)					
Female and Cement	−0.372	0.689	0.158	1.254	<.0001
Female and Wood	−4.893	0.008	0.003	0.017	<.0001
Anti-malarial spraying and use of mosquito nets (ref. Yes & no)					
No and Yes	0.104	1.110	0.898	1.372	0.0319
Gender, main source of drinking water and electricity (ref. Male, protected water & yes)					
Female, tap water and no	0.550	1.734	1.137	2.643	0.0172
Female, unprotected water and no	−1.319	0.267	0.132	0.542	0.0049

Toilet facilities, availability of television, number of rooms per person, main material for walls, number of months the room was sprayed, number of mosquito nets per person, age and family size were found to be significant main effects. In addition to the main effects, five significant two-way interaction terms and one three-way interaction terms was obtained. The two-way interaction terms were: the interaction between main source of drinking water and main material of the room's roof; use of anti-malarial spray and use of mosquito nets; time taken to collect water and floor material; gender and main source of drinking water; gender and main material of the room's floor; and gender and use of anti-malarial spray. Three-way interaction between gender, main source of drinking water and availability of electricity was also significant. Age, family size, toilet facilities, availability of television, number of persons per room, wall material and number of months anti-malarial spray was used were the significant main effects, which were not involved in significant interaction terms (Table 2). Accordingly, the effect of these variables can be directly interpreted using the odds ratio (OR).

Tables 1 and 2 present estimates of socio-economic, demographic and geographic factors on RDT. Based on the result for a unit increase in age, implies a reduction of the odds of a positive malaria test by 3.0% (OR = 0.970, p - value = 0.0001). Furthermore, for a unit increase in family size, the number of persons infected by malaria in the household increased by 5.1% (OR = 1.057, p - value < .0001). Furthermore, compared to households which had no toilet facilities, those with a pit latrine were at lower risk of malaria diagnosis (OR = 0.725, p-value = <.0001) as well as households with flush toilets (OR = 0.552, p - value = <.0001). Households who were using mosquito nets were found to be at a lower risk of malaria compared to the households who were not using mosquito nets (OR = 0.91, p - value = <.0001). Furthermore, for a unit increase in the number of nets, the odds of positive malaria diagnosis test decreases by 54% (OR = 0.46, p - value = <0.0001) for the household.

### Interaction effects

**Figure 1:**
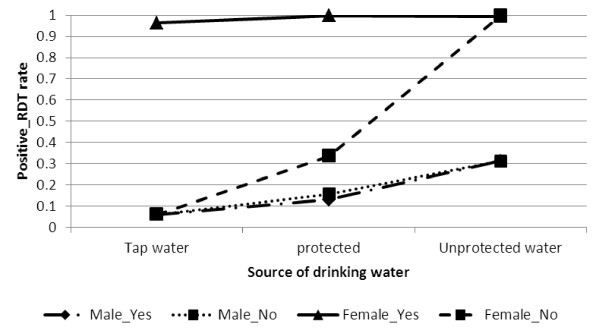
Log odds associated with rapid diagnosis test and gender, source of drinking water with availability of electricity.

The relationship between gender, main source of drinking water and availability of electricity is presented in Figure 1 to indicate the risk of positive malaria RDT is higher for unprotected water use by female respondents. However, for both males and females, positive RDT is low for households using tap water and electricity.

**Figure 2:**
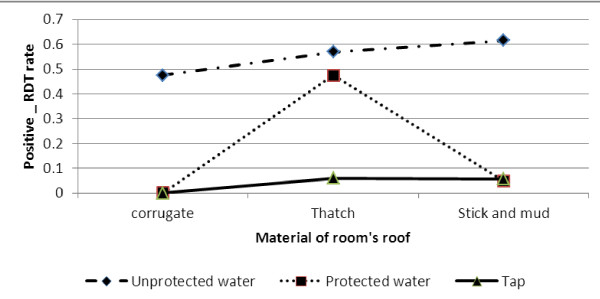
Log odds associated with rapid diagnosis test and material of room's roof with main source of drinking water.

With reference to households that have tap water for drinking and corrugated iron-roofed houses, the risk of positive malaria RDT was significantly lower than for households living in stick and mud-roofed houses and drinking unprotected water (OR = 8.09624, p-value < 0.0001). As Figure 2 indicates, higher positive malaria diagnosis test was found for households that reportedly used unprotected water for drinking.

**Figure 3:**
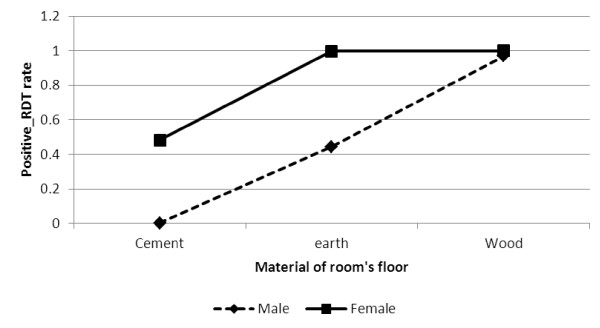
Log odds associated with rapid diagnosis test and gender with material of room's floor.

The OR values for the interaction between gender and main material of the room's floor is given in Figure 3. Based on the result, positive malaria diagnosis test was significantly higher for females than for males who reported that the material of the room’s floor was earth/local dung (OR = 1.358, p - value < .0001) as well as those who reported that the material of the room’s floor was wood (OR = 2.415, p - value < 0.0001). There was however, higher positive malaria diagnosis test found for both males and females who reported that the material of the room’s floor was wood.

**Figure 4:**
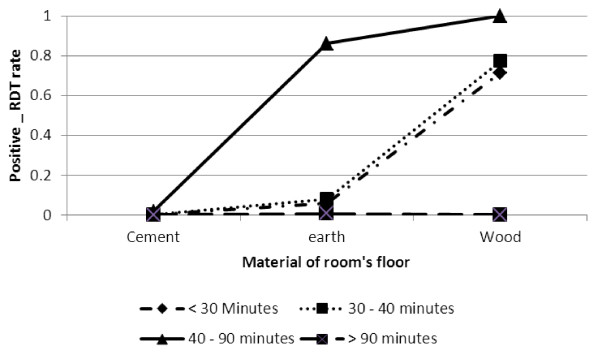
Log odds associated with rapid diagnosis test and material of room's floor with time to collect water.

Positive RDT was significantly higher for respondents living in a room with a wooden or earth/local dung floor than for those living in a room with a cement floor for respondents who took 40–90 minutes to collect water. But, for respondents who took less than 40 minutes to collect water, positive RDT was low (refer Figure 4).

**Figure 5:**
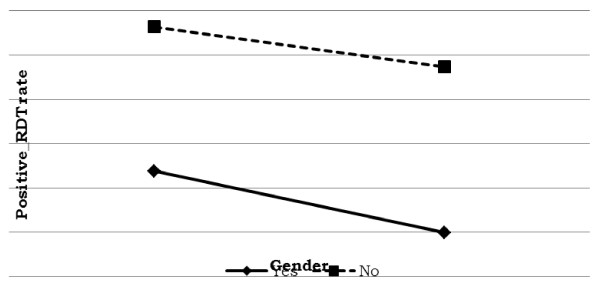
Log odds associated with rapid diagnosis test and anti-malaria spray with gender.

Prevalence of malaria was significantly higher for male than for female respondents who were living in a house treated with anti-malarial spray (refer Figure 5). For both males and females who were living in a house that had not been sprayed, the risk of positive malaria was significantly higher. On the other hand, for males living in a house that had not been treated with anti-malarial spray, the risk of malaria infection for males is more than that of females.

**Figure 6:**
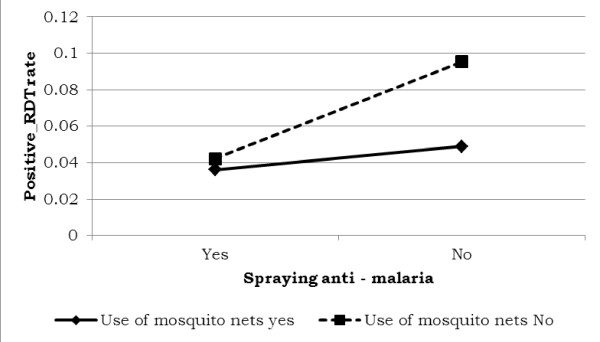
Log odds associated with rapid diagnosis test and use of anti-malaria with use of mosquito nets.

The use of mosquito nets and applying anti-malarial spray to the walls of the house altered the risk of malaria. The risk of malaria was low for individuals who lived in houses that had been sprayed and used malaria nets. It is shown in Figure 6 that the estimated risk of malaria was higher for individuals with no mosquito nets.

## Discussion

The government of Ethiopia has developed strategies related to human resource development, monitoring, and evaluation to control malaria and reduce the hardships it causes. However, the key goals and targets set by the government are aimed at making those areas with historically low malaria transmission, malaria free and a near zero malaria transmission in the remaining malarious areas of the country [35]. Some studies conducted so far have suggested that malaria should be regarded as a disease of the poor or a disease of poverty [36]. This claim can be substantiated by noting the global distribution of malaria where the concentration of the disease is in poorest continents and countries. Being a primary cause of poverty, some studies suggest that a better understanding of the relationships between malaria and poverty is needed to enable the design of coherent and effective policies and tools to tackle the problem. Since poverty is related to socio-economic factors, it is important to identify those factors that are also related to the risk of malaria [37,38].

The present study was conducted based on the 2006 baseline malaria indicator survey in Amhara, Oromiya and Southern Nation Nationalities and People (SNNP) regions of Ethiopia. This survey was a population-based household cluster survey. There were 224 clusters and each cluster consists of 25 households. For this survey, the sampling frame was the rural population of Amhara, Oromiya and SNNP regions. Therefore, the data used for this study was from complex survey. For the statistical analysis, the study used generalized linear model. For this study, gender, age, family size, region, altitude, main source of drinking water, time taken to collect water, toilet facilities, availability of electricity, radio and television, total number of rooms, main material of the room's wall, main material of the room's roof, main material of the room's floor, incidence of anti-malarial spraying in the past 12 months, use of mosquito nets and total number of nets with up to three-way interaction effects were used for the analysis.

Based on these facts, the findings of this study show that the following socio-economic factors are related to malaria risk: construction material of walls, roof and floor of house; main source of drinking water; time taken to collect water; toilet facilities and availability of electricity. Besides socio-economic factors, there are demographic and geographic factors that also had an effect on the risk of malaria. These include gender, age, family size and the region where the respondents lived. In addition to the main effects, there were interactional effects between the socio-economic, demographic and geographic factors that also influenced the risk of malaria. Most notable of these were the interaction between the main source of drinking water and the main construction material of the room's roof; the time taken to collect water and the main construction material of the room's floor; gender and the main source of drinking water; gender and the availability of electricity; gender and the main construction material of the room's floor and finally, interaction between gender, main source of drinking water and the availability of electricity.

From the study, it was observed that residents living in the Amhara region were found to be more at risk of malaria than those living in the SNNP and the Oromiya regions. Similarly, houses that were treated with anti-malarial spray were less likely to be affected by malaria. One of the major challenges in the control of malarial infection was found to be the use of toilet facilities. From the results, it was observed that households with no toilet facilities were more likely to be positive for malaria diagnosis test. Furthermore, positive malaria diagnosis rate decreased with age. But, for households, the risk of malaria increased per unit increase in family size. Generally, malaria parasite prevalence differed between age and gender with the highest prevalence occurring in children and females. The findings of the association between socio-economic factors and malaria prevalence are similar to some of the results from previous studies [39-41]. In addition to this in 1998 and 2000, study was conducted by Ghebreyesus *et al.* and Snow *et al.*[42,43] in Ethiopia and Kenya, respectively. The objectives of the studies were to assess different types of materials used in the construction of walls, roofs and floors of a house. They used generalized linear models, Poisson and logistic models, for their study. Based on their findings, they observed association between any roof, wall and floor material and risk of malaria. Therefore, the finding of this study is similar to the previous results.

This study suggest that having toilet facilities, access to clean drinking water and the use of electricity offers a greater chance of not being positive for malaria diagnosis. Using mosquito nets and spraying anti-malarial treatment on the walls of the house were also found to be a way of reducing the risk of malaria. In addition to this, having a cement floor and corrugated iron roof was found to be one means of reducing the risk of malaria. Based on the study findings, different types of housing have an influence on the risk of malarial transmission with those houses constructed of poor quality materials having an increased risk. Moreover, the presence of particular structural features, such as bricks, that may limit contact with the mosquito vector, also reduces infection. Therefore, the risk of malaria is higher for households in a lower socio-economic bracket than for those that enjoy a higher status and who are able to afford to take measures to reduce the risk of transmission.

This study suggests that with the correct use of mosquito nets, anti-malarial spraying and other preventative measures, coupled with factors such as the number of rooms in a house, the incidence of disease is decreased. However, the study also suggests that the poor are less likely to use these preventative measures to effectively counteract the spread of malaria.

### Ethical clearance

The ethical protocol received approval from the Emory University Institutional Review Board (IRB 1816) and Amhara, Oromiya and SNNPR regional health bureaux. Informed consent was sought in accordance with the tenets of the declaration of Helsinki.

## Abbreviations

FMH: Federal ministry of health of ethiopia; GLM: Generalized linear model; OR: Odds ratio; RDT: Rapid diagnosis test; SNNP: Southern nation nationalities and people; WHO: World health organization.

## Competing interests

The authors declare that they have no competing interests.

## Authors’ contributions

DGA acquired the data, performed the analysis and drafted the manuscript. TTZ and HGM designed the research. All authors discussed the results and implications and commented on the manuscript at all stages. All authors contributed extensively to the work presented in this paper. All authors read and approved the final manuscript.
